# Diagnostic cut-off value of haemoglobin A1c for diabetes mellitus in Harare, Zimbabwe

**DOI:** 10.4102/ajlm.v13i1.2373

**Published:** 2024-04-23

**Authors:** Chido W. Bvumbi, Vinie Kouamou, Ngalulawa Kone, Trust Zaranyika, Lloyd Bowora, Hilda T. Matarira, Raylton P. Chikwati

**Affiliations:** 1 Department of Laboratory Diagnostic and Investigative Sciences, Faculty of Medicine and Health Sciences, University of Zimbabwe, Harare, Zimbabwe; 2 Department of Primary Care and Health Sciences, Internal Medicine Unit, Faculty of Medicine and Health Sciences, University of Zimbabwe, Harare, Zimbabwe; 3 Biomedical Research and Training Institute, Harare, Zimbabwe; 4 Department of Chemical Pathology, Faculty of Health Sciences, University of the Witwatersrand, Johannesburg, South Africa; 5 Department of Chemical Pathology, National Health Laboratory Service, Johannesburg, South Africa; 6 Sydney Brenner Institute for Molecular Bioscience, Faculty of Health Sciences, University of the Witwatersrand, Johannesburg, South Africa

**Keywords:** glycated haemoglobin, diabetes mellitus, fasting plasma glucose, receiver operating characteristics curves, Zimbabwe

## Abstract

Very little is known about the diagnostic performance of the American Diabetes Association glycated haemoglobin (HbA1c) cut-off of 6.5% in resource-limited settings. This study, conducted between February 2023 and May 2023, aimed to determine the optimal HbA1c cut-off for the diagnosis of diabetes mellitus by measuring HbA1c and fasting plasma glucose levels in 120 adults attending care at a tertiary hospital in Harare, Zimbabwe. The optimal HbA1c cut-off was 6.1% and glucose levels were strongly correlated with HbA1c values. The prevalence of diabetes mellitus was higher (28.3%) at our derived HbA1c cut-off than with the American Diabetes Association criterion (21.6%).

**What this study adds:**

This study highlights the need for population-specific cut-off HbA1c values in the diagnosis of diabetes mellitus.

## Introduction

The prevalence of diabetes mellitus (DM) in Zimbabwe has increased from 0.4% (95% confidence interval [CI]: 0.0–1.9) before 1980, to 5.7% (95% CI: 3.3–8.6) after 1980.^1^ While the reasons behind these changes are multifactoral, a key factor could be the transition in the diagnostic criteria of DM from urine to blood testing. From the eight studies evaluated, three used urine for glucose testing and were all conducted before 1980.^1^ When comparing the prevalence between these studies, it is apparent that the transition from urine to blood testing had an impact on the observed increase. It is therefore important to evaluate the diagnostic criteria in this population to improve diabetic care.

Currently, a glycated haemoglobin (HbA1c) of 6.5% is used for diagnosing DM, as guided by the American Diabetes Association (ADA).^2^ However, the diagnostic cut-off value of HbA1c has been shown to vary across different races and ethnicities.^3^ Studies have shown that HbA1c levels are higher in African Americans, African Brazilians, British African Caribbeans, Asians and Latinos than in populations of European ancestry.^4,5^ Given this evidence, a universal HbA1c cut-off may underdiagnose DM in some populations. We therefore aimed to determine an optimal HbA1c cut-off value for DM in a black Zimbabwean population of African ancestry.

## Methods

### Ethical considerations

This study was approved by the Joint Research Ethics Committee of the University of Zimbabwe (JREC 48/2023) and the Parirenyatwa Group of Hospital’s Clinical Director. Informed written consent was obtained from all participants. To protect privacy and confidentiality, all collected blood specimens were de-identified to ensure that no individual could be linked to the study information. All data were captured on a password-protected Excel spreadsheet (Microsoft Corp., Redmond, Washington, United States) and printed results were stored in a cabinet only accessible to the study investigators.

### Study design

In this cross-sectional study, we recruited adults (≥ 18 years) from Harare, Zimbabwe, with a body mass index ≥ 25 kg/m^2^. Recruitment of study participants was done from 15 February 2023 to 14 May 2023 at the Diabetic Clinic, Parirenyatwa Group of Hospitals, a tertiary hospital. A minimum sample size of 119 participants was calculated using the Dobson formula. Participants who self-reported pregnancy or had anaemia, renal disorders or haemoglobinopathies based on a previous diagnosis by a healthcare practitioner were excluded.

### Specimen processing

Blood specimens were collected by trained nurses and only used for this study. From each participant, approximately 8 mL of blood were collected after an 8 h overnight fast. Participants were advised to have their dinner before midnight and not ingest any food or drink, except for water, until the next day when blood was drawn. Blood specimens were collected in sodium fluoride- and ethylenediamine tetraacetic acid-containing vacutainer tubes (Becton, Dickinson and Company, Plymouth, United Kingdom). All blood specimens were transported from the clinic to the Interpath Medical Laboratories, where they were processed, analysed and stored.

At the laboratory, blood specimens in sodium fluoride tubes were immediately centrifuged at 3500 revolutions per minute for 5 min (Yingtai Instrument, Changsha City, China). Testing of fasting plasma glucose (FPG) was done within 2 h from blood collection. Ethylenediamine tetraacetic acid blood specimens were stored at 2 °C – 8 °C before transportation on dry ice to the National Health Laboratory Service in Johannesburg, South Africa, for HbA1c measurement.

### Anthropometric variables

Body weight was measured using a 120 kg scale (MCP Camry, Medicare Products Inc., New Delhi, India). Participants were asked to wear light clothing before standing on the scale. Standing height was measured using a wooden board which had a sliding headpiece (United Nations Children’s Fund, New York, New York, United States). Participants were asked to maintain a straight posture against the wall before a reading was taken. Body mass index was calculated as a ratio of the measured weight in kg and height in m^2^.

### Fasting plasma glucose

Fasting plasma glucose levels were measured using the hexokinase methodology on a Mindray analyser (Mindray BS200, Shenzhen, China). Mindray reagents, calibrators, and quality control materials were used. Diabetes mellitus was defined as a FPG ≥ 7.0 mmol/L.^2^

### Glycated haemoglobin

HbA1c levels were measured in ethylenediamine tetraacetic acid whole blood using a Bio-Rad High-Performance Liquid Chromatography analyser, reagents, calibrators, and quality control materials (Bio-Rad VARIANT II, Montreal, Quebec, Canada).

### Statistical analyses

All statistical analyses were performed using Stata 16.1 (StataCorp LP, College Station, Texas, United States). Continuous variables were expressed as mean ± standard deviation if data followed a Gaussian distribution, or median and interquartile range (IQR) if data were skewed. Continuous variables were compared using the Student’s t-test and Mann-Whitney U test, as appropriate. Pearson correlation was used to assess the relationship between FPG and HbA1c levels. Statistical significance was set at *p* < 0.05. Receiver operator characteristic curves were plotted to identify the diagnostic cut-off for HbA1c using the highest Youden’s J index.^6^ Sensitivity and specificity were calculated for both the derived and ADA HbA1c cut-off values.

## Results

### Characteristics of study population

We recruited a total of 120 participants, comprising 62 women and 58 men, from a pool of 138 eligible participants. Non-participation was primarily due to the inability to commute to the study clinic and failure to provide a fasting blood specimen. Their mean (± standard deviation) age was 43.2 (±14.0) years. Median (IQR) body mass index was 29.0 (26.5–31.3) kg/m^2^. Body mass index was higher in women than men. No other differences were observed (Table 1).

**TABLE 1:** Characteristics of study participants, Harare, Zimbabwe, February 2023 to May 2023.

Variable	Total (*N* = 120)	Women (*n* = 68)	Men (*n* = 52)	*p*
**Age (years)**	-	-	-	0.170
Mean ± standard deviation	43.2 ± 14.0	44.7 ± 12.8	41.2 ± 15.2	-
**BMI (kg/m^2^)**	-	-	-	0.001
Median	29.0	30.1	27.7	-
IQR	26.5–31.3	28.0–32.6	26.1–30.1	-
**FPG (mmol/L)**	-	-	-	0.795
Median	5.21	5.12	5.29	-
IQR	4.35–7.00	4.33–7.03	4.45–6.99	-
**HbA1c (%)**	-	-	-	0.562
Median	5.55	5.50	5.60	-
IQR	5.05–6.30	5.00–6.25	5.15–6.35	-

Note: Data presented as mean ± standard deviation or median and interquartile range (IQR).

BMI, body mass index; FPG, fasting plasma glucose; HbA1c, glycated haemoglobin; IQR, interquartile range.

### Fasting plasma glucose and HbA1c

**FIGURE 1:**
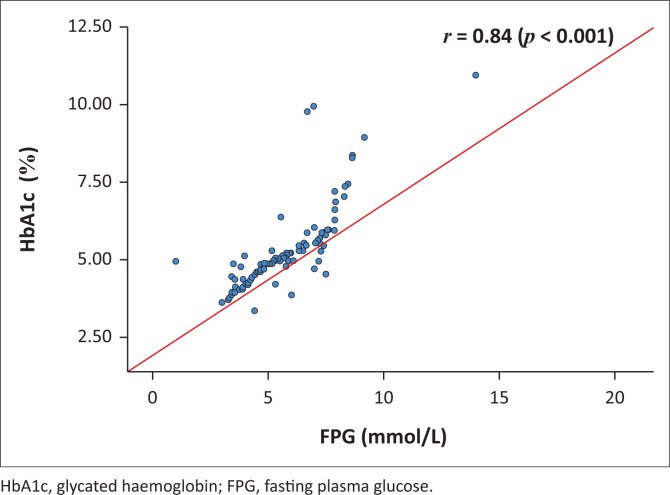
Correlation between HbA1c and FPG, Harare, Zimbabwe, February 2023 to May 2023.

The median (IQR) FPG levels were 5.21 (4.35 – 7.00) mmol/L and HbA1c, 5.55% (5.05% – 6.30%) (Table 1). When the ADA criterion (FPG ≥ 7.0 mmol/L) was applied, 31 (26.0%) participants had DM. Their median (IQR) FPG levels were 7.50 (7.23 – 8.12) mmol/L and HbA1c, 6.80% (6.35% – 8.40%) (data not shown). Furthermore, there was a linear relationship between FPG and HbA1c levels (*r* = 0.84, *p* < 0.001) (Figure 1).

**FIGURE 2:**
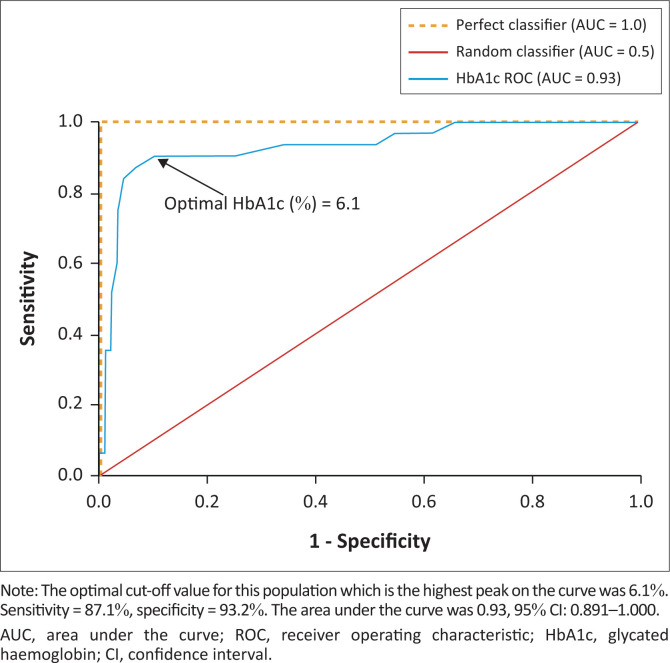
Receiver operating characteristic curve for the determination of the optimal cut-off value of HbA1c for the diagnosis of diabetes mellitus in all study participants, Harare, Zimbabwe, February 2023 to May 2023.

**FIGURE 3:**
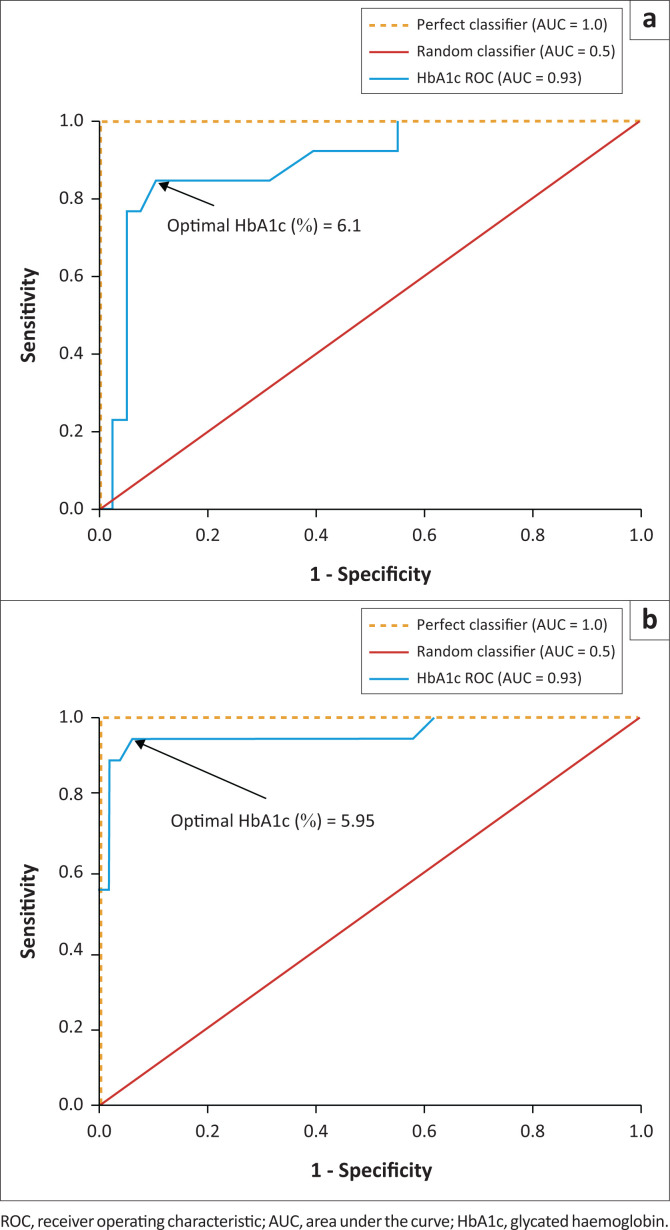
Receiver operating characteristic curves for optimal HbA1c cut-off values for the diagnosis of diabetes mellitus in men and women, Harare, Zimbabwe, February 2023 – May 2023. (a) The optimal cut-off value for men was 6.1%. Sensitivity = 84.6%, specificity = 89.5%. The area under the curve was 0.89, 95% confidence interval (CI): 0.783–0.994. (b) The optimal cut-off value for women was 5.95%. Sensitivity = 94.4%, specificity = 94%. The area under the curve was 0.96, 95% CI: 0.810–1.000.

For all participants, the optimal HbA1c cut-off for DM was 6.10% (Figure 2). When stratified according to sex, the optimal HbA1c cut-off was unchanged in men, at 6.10%, but lower in women, at 5.95% (Figure 3).

### Diagnostic performance

The derived HbA1c cut-off of 6.1% had a higher sensitivity (87.1%) than the ADA criterion (71.0%) (Online Supplementary Table 1). However, specificity was slightly higher in the ADA criterion (95.5% vs 93.2%). The prevalence of DM was higher (28.3%) when the 6.1% cut-off was applied, but lower with the ADA criterion (21.6%) (Online Supplementary Table 1).

## Discussion

Our results showed that a 6.1% HbA1c cut-off had high sensitivity and specificity in black Zimbabwean participants with body mass index ≥ 25 kg/m^2^. When we applied the ADA criterion, the sensitivity decreased, and we identified 6.7% fewer diabetics.

In support of our findings, a meta-analysis from 2022 including seven African studies showed that the sensitivity for HbA1c in the diagnosis of DM was highest (74.9%) at 6.0%.^7^ Two global meta-analyses showed that the 6.5% cut-off was associated with lower sensitivity (59.4% and 52.7%).^8,9^ Other studies from South Africa (6.1%),^10^ India (6.1%),^11^ and Haiti (6.3%)^12^ have also shown lower HbA1c cut-offs than the ADA criterion.

Our findings and others’ indicate that the optimal Hb1Ac cut-off differs from the ADA criterion. Some factors, such as Hb1Ac analytical methods and haemoglobin structure, may contribute to these differences.^13^ Furthermore, different inclusion criteria may contribute to heterogeneity between studies.^14,15^

We also observed a positive linear relationship between FPG and HbA1c levels. These findings are consistent with a meta-analysis conducted in 2015 which reported a similar trend (*r* = 0.74, *p* < 0.001).^16^

The slightly lower HbA1c cut-off found in our study, of 5.95% in women compared to 6.10% in men, has not been reported previously. However, studies have shown that black women of African ancestry have lower hepatic clearance of insulin, differential ß-cell responsiveness and therefore higher mean blood glucose levels than white women of European ancestry.^17,18^ However, there is scarcity of data comparing black women and black men.

Strengths of our study include using the high-performance liquid chromatography method and recruiting from one urban population, where lifestyle and environmental factors were likely to be homogenous. In addition, all specimens were collected within the same weather season (autumn), thus the seasonal influence on HbA1c was minimal.

### Limitations

Limitations of our study include its small sample size, and sampling from a tertiary clinic; therefore, findings may not be generalisable. Additionally, due to limited resources, we did not have specific symptomology, such as diabetic retinopathy, to confirm DM. We only collected data on body mass index, but not other risk factors that include family history, dietary intake, alcohol consumption, and hypertension. These could have been valuable in assessing the impact of the diagnostic performance of HbA1c against FPG. Another limitation was that we relied on ordered fasting which could not be standardised since participants would present at the study clinic after an overnight fast. It is possible that not all participants were compliant to the instructions given, as some could have misinterpreted that fasting only applies to meals, not beverages and sweets before the test. Furthermore, we did not perform the oral glucose tolerance test, which is known for its higher sensitivity and specificity in the diagnosis of DM.

### Conclusion

This study demonstrates a higher diagnostic performance of an HbA1c cut-off value of 6.1% in a black population from Harare, Zimbabwe, and that the ADA cut-off was not optimal for this population. More studies are warranted to confirm these findings in a larger population with better representation of geographical location and more standardised methods of collecting fasting specimens.
